# *N*-(2-Thienylmethyl­ene)-2-(2-{[2-(2-thienylmethyl­eneamino)phen­yl]sulfan­yl}ethyl­sulfan­yl)aniline

**DOI:** 10.1107/S1600536809024404

**Published:** 2009-06-27

**Authors:** Ali Kakanejadifard, Vahid Amani

**Affiliations:** a Department of Chemistry, Faculty of Science, Lorestan University, Khorramabad, Iran; b Department of Chemistry, Islamic Azad University, Shahr-e-Rey Branch, Tehran, Iran

## Abstract

The asymmetric unit of the title compound, C_24_H_20_N_2_S_4_, contains one half-mol­ecule: a crystallographic centre of inversion is located at the mid-point of the two central C atoms. The thio­phene ring is oriented at a dihedral angle of 60.64 (3)° with respect to the benzene ring. In the crystal structure, π–π contacts between thio­phene rings [centroid–centroid distance = 3.581 (1) Å] may stabilize the structure. A weak C—H⋯π inter­action is also present.

## Related literature

For related structures, see: Dharaa *et al.* (2005 ▶); Gok & Demirbas (1989 ▶); Kakanejadifard *et al.* (2007 ▶); Kakanejadifard & Amani (2008 ▶); Morshedi *et al.* (2009 ▶); Rajsekhar *et al.* (2002 ▶, 2004 ▶); Taylor *et al.* (2008 ▶). For bond-length data, see: Allen *et al.* (1987 ▶).
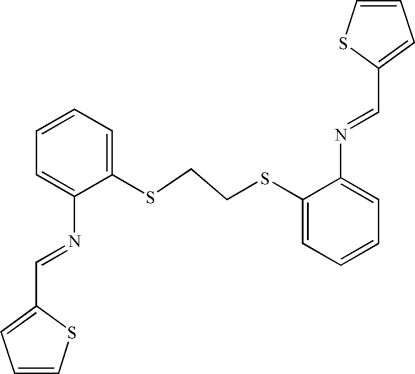


## Experimental

### 

#### Crystal data

C_24_H_20_N_2_S_4_
*M* *_r_* = 464.66
Monoclinic, 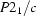

*a* = 11.179 (5) Å
*b* = 7.730 (4) Å
*c* = 12.608 (6) Å
β = 91.899 (12)°
*V* = 1088.9 (9) Å^3^
*Z* = 2
Mo *K*α radiation
μ = 0.45 mm^−1^
*T* = 100 K
0.30 × 0.20 × 0.15 mm

#### Data collection

Bruker Kappa APEXII CCD diffractometer
Absorption correction: multi-scan (*SADABS*; Bruker, 2005 ▶) *T*_min_ = 0.895, *T*_max_ = 0.930
12880 measured reflections
2899 independent reflections
2569 reflections with *I* > 2/s(*I*)
*R*_int_ = 0.029

#### Refinement

*R*[*F* ^2^ > 2σ(*F* ^2^)] = 0.029
*wR*(*F* ^2^) = 0.079
*S* = 1.00
2899 reflections
136 parameters
H-atom parameters constrained
Δρ_max_ = 0.34 e Å^−3^
Δρ_min_ = −0.26 e Å^−3^

### 

Data collection: *APEX2* (Bruker, 2005 ▶); cell refinement: *SAINT* (Bruker, 2005 ▶); data reduction: *SAINT*; program(s) used to solve structure: *SHELXTL* (Sheldrick, 2008 ▶); program(s) used to refine structure: *SHELXTL*; molecular graphics: *SHELXTL* software used to prepare material for publication: *SHELXTL*.

## Supplementary Material

Crystal structure: contains datablocks I, global. DOI: 10.1107/S1600536809024404/hk2716sup1.cif

Structure factors: contains datablocks I. DOI: 10.1107/S1600536809024404/hk2716Isup2.hkl

Additional supplementary materials:  crystallographic information; 3D view; checkCIF report

## Figures and Tables

**Table 1:** Hydrogen-bond geometry (Å, °)

*D*—H⋯*A*	*D*—H	H⋯*A*	*D*⋯*A*	*D*—H⋯*A*
C10—H10*A*⋯*Cg*1^i^	0.95	2.80	3.740 (3)	171

Symmetry code: (i) 
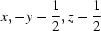
. *Cg*1 is centroid of the ring C2–C7 ring.

**Fig. 1.**
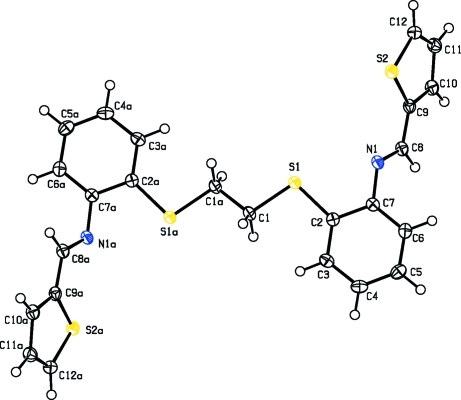
The molecular structure of the title molecule, with the atom-numbering scheme. Displacement ellipsoids are drawn at the 50% probability level [symmetry code: (a) 1 - x, -y, 2 -z].

**Fig. 2.**
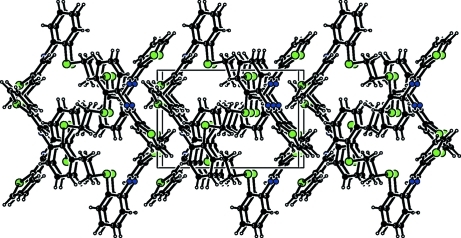
A partial packing diagram.

## supplementary crystallographic information

### Comment

There are several examples of N_2_S_2_ Schiff bases type of adducts which exist as anti configuration. For 2-[2-(2-aminophenylthio)benzeneamine] adduct see: (Gok & Demirbas, 1989; Dharaa *et al.*, 2005; Kakanejadifard *et al.*, 2007; Kakanejadifard & Amani, 2008). For N_2_S_2_ Schiff bases adduct see: (Rajsekhar *et al.*, 2002; Taylor *et al.*, 2008; Morshedi *et al.*, 2009). For N_2_O_2_S_2_ Schiff bases adduct see: (Rajsekhar *et al.*, 2004). We report herein the synthesis and crystal structure of the title compound.

The asymmetric unit of the title compound, (Fig. 1), contains one-half molecule. A crystallographic centre of inversion is located at the midpoint between the two central C atoms. The bond lengths (Allen *et al.*, 1987) and angles are within normal ranges. Rings A (C2-C7) and B (S2/C9-C12) are, of course, planar and they are oriented at a dihedral angle of 60.64 (3)°.

In the crystal structure, the π–π contact between the thiophene rings, Cg2—Cg2^i^, [symmetry code: (i) -x, -y, 1 - z, where Cg2 is centroid of the ring B (S2/C9-C12)] may stabilize the structure, with centroid-centroid distance of 3.581 (1) Å. There also exits a weak C—H···π interaction (Table 1).

### Experimental

For the preparation of the title compound, a solution of thiophencarbaldehyde (20 mmol) was added dropwise to a solution of 2-[2-(2-aminophenylthio)benzeneamine] (2.76 g, 10 mmol) in absolute ethanol (25 ml) with stirring in 10 min at room temperature. The mixture was stirred and heated to reflux for 5 h. The product was filtered and crystallized from CH_3_CN (yield; 45%, m.p. 398-399 K).

### Refinement

H atoms were positioned geometrically with C-H = 0.95 and 0.99 Å for aromatic and methylene H atoms, respectively, and constrained to ride on their parent atoms, with *U*_iso_(H) = *1.2U*_eq_(C).

### Crystal data

**Table:**

C_24_H_20_N_2_S_4_	*F*(000) = 484
*M**_r_* = 464.66	*D*_x_ = 1.417 Mg m^−^^3^
Monoclinic, *P*2_1_/*c*	Mo *K*α radiation, λ = 0.71073 Å
Hall symbol: -P 2ybc	Cell parameters from 887 reflections
*a* = 11.179 (5) Å	θ = 3–30°
*b* = 7.730 (4) Å	µ = 0.45 mm^−^^1^
*c* = 12.608 (6) Å	*T* = 100 K
β = 91.899 (12)°	Prism, yellow
*V* = 1088.9 (9) Å^3^	0.30 × 0.20 × 0.15 mm
*Z* = 2	

### Data collection

**Table:**

Bruker Kappa APEXII CCD diffractometer	2899 independent reflections
Radiation source: fine-focus sealed tube	2569 reflections with *I* > 2/s(*I*)
graphite	*R*_int_ = 0.029
φ and ω scans	θ_max_ = 29.0°, θ_min_ = 1.8°
Absorption correction: multi-scan (*SADABS*; Bruker, 2005)	*h* = −15→15
*T*_min_ = 0.895, *T*_max_ = 0.930	*k* = −10→10
12880 measured reflections	*l* = −17→17

### Refinement

**Table:**

Refinement on *F*^2^	Primary atom site location: structure-invariant direct methods
Least-squares matrix: full	Secondary atom site location: difference Fourier map
*R*[*F*^2^ > 2σ(*F*^2^)] = 0.029	Hydrogen site location: inferred from neighbouring sites
*wR*(*F*^2^) = 0.079	H-atom parameters constrained
*S* = 1.00	*w* = 1/[σ^2^(*F*_o_^2^) + (0.0441*P*)^2^ + 0.43*P*] where *P* = (*F*_o_^2^ + 2*F*_c_^2^)/3
2899 reflections	(Δ/σ)_max_ = 0.001
136 parameters	Δρ_max_ = 0.34 e Å^−^^3^
0 restraints	Δρ_min_ = −0.26 e Å^−^^3^

### Special details

**Table:**

Geometry. All e.s.d.'s (except the e.s.d. in the dihedral angle between two l.s. planes) are estimated using the full covariance matrix. The cell e.s.d.'s are taken into account individually in the estimation of e.s.d.'s in distances, angles and torsion angles; correlations between e.s.d.'s in cell parameters are only used when they are defined by crystal symmetry. An approximate (isotropic) treatment of cell e.s.d.'s is used for estimating e.s.d.'s involving l.s. planes.
Refinement. Refinement of *F*^2^ against ALL reflections. The weighted *R*-factor *wR* and goodness of fit *S* are based on *F*^2^, conventional *R*-factors *R* are based on *F*, with *F* set to zero for negative *F*^2^. The threshold expression of *F*^2^ > σ(*F*^2^) is used only for calculating *R*-factors(gt) *etc*. and is not relevant to the choice of reflections for refinement. *R*-factors based on *F*^2^ are statistically about twice as large as those based on *F*, and *R*- factors based on ALL data will be even larger.

### Fractional atomic coordinates and isotropic or equivalent isotropic displacement parameters (Å^2^)

**Table:**

	*x*	*y*	*z*	*U*_iso_*/*U*_eq_	
S1	0.63766 (3)	−0.06436 (4)	0.88963 (2)	0.01981 (9)	
S2	0.96427 (3)	0.15452 (4)	0.67870 (2)	0.01990 (9)	
N1	0.79244 (9)	−0.14058 (14)	0.72259 (8)	0.0188 (2)	
C1	0.51924 (11)	−0.09011 (16)	0.98300 (10)	0.0199 (2)	
H1A	0.4507	−0.1525	0.9492	0.024*	
H1B	0.5485	−0.1574	1.0454	0.024*	
C2	0.64682 (11)	−0.27273 (16)	0.83345 (10)	0.0178 (2)	
C3	0.58160 (11)	−0.41715 (17)	0.86395 (10)	0.0212 (3)	
H3A	0.5258	−0.4071	0.9188	0.025*	
C4	0.59782 (12)	−0.57566 (17)	0.81445 (11)	0.0232 (3)	
H4A	0.5528	−0.6732	0.8357	0.028*	
C5	0.67894 (12)	−0.59283 (18)	0.73454 (11)	0.0238 (3)	
H5A	0.6912	−0.7024	0.7025	0.029*	
C6	0.74233 (12)	−0.44926 (17)	0.70128 (10)	0.0216 (3)	
H6A	0.7970	−0.4605	0.6456	0.026*	
C7	0.72613 (10)	−0.28888 (16)	0.74913 (9)	0.0180 (2)	
C8	0.79459 (11)	−0.09360 (17)	0.62510 (10)	0.0196 (2)	
H8A	0.7488	−0.1565	0.5733	0.024*	
C9	0.86458 (11)	0.05200 (17)	0.59218 (10)	0.0188 (2)	
C10	0.86686 (12)	0.12347 (18)	0.49235 (10)	0.0213 (3)	
H10A	0.8185	0.0843	0.4338	0.026*	
C11	0.94924 (12)	0.26160 (18)	0.48653 (10)	0.0234 (3)	
H11A	0.9619	0.3261	0.4237	0.028*	
C12	1.00852 (12)	0.29220 (17)	0.58100 (10)	0.0223 (3)	
H12B	1.0675	0.3797	0.5914	0.027*	

### Atomic displacement parameters (Å^2^)

**Table:**

	*U*^11^	*U*^22^	*U*^33^	*U*^12^	*U*^13^	*U*^23^
S1	0.01936 (16)	0.01889 (16)	0.02154 (16)	−0.00077 (11)	0.00610 (11)	−0.00011 (11)
S2	0.02172 (16)	0.02322 (17)	0.01469 (15)	−0.00207 (11)	−0.00047 (11)	−0.00144 (11)
N1	0.0163 (5)	0.0226 (5)	0.0174 (5)	−0.0002 (4)	0.0025 (4)	−0.0003 (4)
C1	0.0180 (6)	0.0221 (6)	0.0199 (6)	0.0015 (5)	0.0049 (4)	0.0027 (5)
C2	0.0168 (5)	0.0188 (6)	0.0178 (5)	0.0017 (4)	−0.0008 (4)	0.0010 (4)
C3	0.0190 (6)	0.0226 (6)	0.0221 (6)	−0.0004 (5)	0.0013 (5)	0.0045 (5)
C4	0.0226 (6)	0.0196 (6)	0.0270 (6)	−0.0027 (5)	−0.0036 (5)	0.0044 (5)
C5	0.0239 (6)	0.0210 (6)	0.0261 (6)	0.0016 (5)	−0.0053 (5)	−0.0026 (5)
C6	0.0196 (6)	0.0251 (6)	0.0200 (6)	0.0021 (5)	−0.0005 (5)	−0.0024 (5)
C7	0.0160 (5)	0.0210 (6)	0.0170 (5)	−0.0001 (4)	−0.0013 (4)	0.0014 (4)
C8	0.0173 (5)	0.0240 (6)	0.0176 (6)	0.0002 (5)	0.0007 (4)	−0.0011 (5)
C9	0.0173 (5)	0.0229 (6)	0.0163 (5)	0.0004 (4)	0.0001 (4)	−0.0020 (5)
C10	0.0220 (6)	0.0259 (6)	0.0159 (6)	0.0006 (5)	−0.0010 (4)	0.0000 (5)
C11	0.0277 (6)	0.0230 (6)	0.0198 (6)	0.0008 (5)	0.0034 (5)	0.0037 (5)
C12	0.0247 (6)	0.0193 (6)	0.0232 (6)	−0.0022 (5)	0.0032 (5)	0.0002 (5)

### Geometric parameters (Å, °)

**Table:**

S1—C2	1.7639 (15)	C4—H4A	0.9500
S1—C1	1.8114 (14)	C5—C6	1.389 (2)
S2—C12	1.7132 (15)	C5—H5A	0.9500
S2—C9	1.7261 (14)	C6—C7	1.3932 (19)
N1—C8	1.2827 (17)	C6—H6A	0.9500
N1—C7	1.4114 (17)	C8—C9	1.4396 (18)
C1—C1^i^	1.524 (3)	C8—H8A	0.9500
C1—H1A	0.9900	C9—C10	1.3757 (18)
C1—H1B	0.9900	C10—C11	1.414 (2)
C2—C3	1.3944 (18)	C10—H10A	0.9500
C2—C7	1.4122 (17)	C11—C12	1.3644 (19)
C3—C4	1.3897 (19)	C11—H11A	0.9500
C3—H3A	0.9500	C12—H12B	0.9500
C4—C5	1.384 (2)		
			
C2—S1—C1	102.36 (6)	C5—C6—C7	120.35 (12)
C12—S2—C9	91.53 (7)	C5—C6—H6A	119.8
C8—N1—C7	118.94 (11)	C7—C6—H6A	119.8
C1^i^—C1—S1	107.55 (11)	C6—C7—N1	122.90 (11)
C1^i^—C1—H1A	110.2	C6—C7—C2	119.91 (12)
S1—C1—H1A	110.2	N1—C7—C2	117.03 (11)
C1^i^—C1—H1B	110.2	N1—C8—C9	121.68 (12)
S1—C1—H1B	110.2	N1—C8—H8A	119.2
H1A—C1—H1B	108.5	C9—C8—H8A	119.2
C3—C2—C7	118.94 (12)	C10—C9—C8	127.19 (12)
C3—C2—S1	125.63 (10)	C10—C9—S2	111.26 (10)
C7—C2—S1	115.42 (9)	C8—C9—S2	121.50 (10)
C4—C3—C2	120.32 (12)	C9—C10—C11	112.50 (12)
C4—C3—H3A	119.8	C9—C10—H10A	123.7
C2—C3—H3A	119.8	C11—C10—H10A	123.7
C5—C4—C3	120.66 (12)	C12—C11—C10	112.59 (12)
C5—C4—H4A	119.7	C12—C11—H11A	123.7
C3—C4—H4A	119.7	C10—C11—H11A	123.7
C4—C5—C6	119.75 (13)	C11—C12—S2	112.12 (10)
C4—C5—H5A	120.1	C11—C12—H12B	123.9
C6—C5—H5A	120.1	S2—C12—H12B	123.9
			
C2—S1—C1—C1^i^	−166.07 (12)	S1—C2—C7—C6	178.23 (9)
C1—S1—C2—C3	−4.13 (13)	C3—C2—C7—N1	−178.52 (11)
C1—S1—C2—C7	174.52 (9)	S1—C2—C7—N1	2.73 (14)
C7—C2—C3—C4	2.25 (19)	C7—N1—C8—C9	−177.31 (11)
S1—C2—C3—C4	−179.14 (10)	N1—C8—C9—C10	−174.31 (13)
C2—C3—C4—C5	0.2 (2)	N1—C8—C9—S2	8.43 (18)
C3—C4—C5—C6	−1.9 (2)	C12—S2—C9—C10	−0.04 (10)
C4—C5—C6—C7	1.1 (2)	C12—S2—C9—C8	177.62 (11)
C5—C6—C7—N1	176.60 (12)	C8—C9—C10—C11	−177.69 (12)
C5—C6—C7—C2	1.38 (19)	S2—C9—C10—C11	−0.20 (15)
C8—N1—C7—C6	53.92 (17)	C9—C10—C11—C12	0.42 (17)
C8—N1—C7—C2	−130.73 (13)	C10—C11—C12—S2	−0.44 (15)
C3—C2—C7—C6	−3.02 (18)	C9—S2—C12—C11	0.28 (11)

Symmetry codes: (i) −*x*+1, −*y*, −*z*+2.

### Hydrogen-bond geometry (Å, °)

**Table:**

*D*—H···*A*	*D*—H	H···*A*	*D*···*A*	*D*—H···*A*
C10—H10A···Cg1^ii^	0.95	2.80	3.740 (3)	171

Symmetry codes: (ii) *x*, −*y*−1/2, *z*−1/2.
